# Awkward Choreographies from Cancer's Margins: Incommensurabilities of Biographical and Biomedical Knowledge in Sexual and/or Gender Minority Cancer Patients’ Treatment

**DOI:** 10.1007/s10912-018-9542-0

**Published:** 2018-11-29

**Authors:** Mary K. Bryson, Evan T. Taylor, Lorna Boschman, Tae L. Hart, Jacqueline Gahagan, Genevieve Rail, Janice Ristock

**Affiliations:** 1 Department of Language and Literacy Education, Faculty of Education, University of British Columbia, Vancouver, BC Canada; 2 Department of Language and Literacy Education, UBC, Vancouver, BC Canada; 3 Department of Psychology, Ryerson University, Toronto, ON Canada; 4 School of Health and Human Performance, Dalhousie University, Halifax, NS Canada; 5 Simone de Beauvoir Institute, Concordia University, Montréal, QC, Canada; 6 Women’s and Gender Studies, University of Manitoba, Winnipeg, MB Canada

**Keywords:** Illness narratives, Biographical knowledge, Choreography, Access to knowledge, Cancer, Sexualities, Genders, LGBT, Transgender, Health disparities, Equity

## Abstract

Canadian and American population-based research concerning sexual and/or gender minority populations provides evidence of persistent breast and gynecologic cancer-related health disparities and knowledge divides. The *Cancer's Margins* research investigates the complex intersections of sexual and/or gender marginality and incommensurabilities and improvisation in engagements with biographical and biomedical cancer knowledge. The study examines how sexuality and gender are intersectionally constitutive of complex biopolitical mappings of cancer health knowledge that shape knowledge access and its mobilization in health and treatment decision-making. Interviews were conducted with a diverse group (*n*=81) of sexual and/or gender minority breast or gynecologic cancer patients. The LGBQ//T2 cancer patient narratives we have analyzed document in fine grain detail how it is that sexual and/or gender minority cancer patients punctuate the otherwise lockstep assemblage of their cancer treatment decision-making with a persistent engagement in creative attempts to resist, thwart and otherwise manage the possibility of discrimination and likewise, the probability of institutional erasure in care settings. Our findings illustrate the demands that cancer places on LGBQ//T2 patients to choreograph access to, and mobilization of knowledge and care, across significantly distinct and sometimes incommensurable systems of knowledge.

He said a single mastectomy with reconstruction would be best. The reconstructive surgery best suited to my body was a latissimus flap… He explained how he would carve apart the largest muscle in my back, and, with one end of the muscle connected to its blood supply, tunnel the loose end (the flap) through my body and under my arm until it reached the empty socket on my chest where my breast had been. Then he would pull the flap of muscle up and over a silicone implant. I pictured a steak laid over a tennis ball.

“Isn’t that muscle doing something?” I asked.

“Most women hardly miss it,” he said, his eyes on his notes.

Most women?

“Most women just want to look normal in clothes,” he added, still not looking up.

Normal?

“Is there any other way? Something that doesn’t involve implants and rearranging muscles?”

The question sounded childish. Why was I so peevish, so maladaptive that I couldn’t get with the program?

Catherine Guthrie, *Flat: Reclaiming My Body From Breast Cancer* (2018, 81-82)

## Context for study

### Cancer on the margins

Recent public health and policy research in the Global North affirms an urgent consensus that “vulnerable” populations are not well served by the current generation, translation, and mobilization of cancer knowledge, especially in light of related, longstanding health disparities (Burkhalter et al. 2016; Polite et al. 2017). North American population-based surveys among sexual and/or gender minority populations provide evidence of persistent breast and gynecologic cancer-related health disparities and knowledge divides (Cathcart-Rake 2018; Gibson et al. 2017; Peitzmeier et al. 2014; Sinding, Grassau and Barnoff 2006).

Sexuality and gender are distinct and yet intersectionally linked aspects of health and wellbeing (Institute of Medicine 2011, Meyer 2012). The most common acronym used to identify distinct groups of sexual and gender minority groups is: LGBQ//T2^1^ (lesbian, gay, bisexual, queer // transgender and Two-Spirit). Research consistently shows QLB women are less likely to have a regular physician, to participate in breast and gynecologic cancer screening, and more likely to express dissatisfaction with HCPs during cancer treatment (Fobair et al. 2001; Grindel et al. 2006; Tjepkema 2008). Relatedly, research on trans and/or gender nonconforming populations reports that over 50% of trans people report negative health care experiences, that 21% avoided emergency health care as a result (Bauer et al. 2014), and have less access to breast and gynecologic cancer screening (Scheim & Bauer 2013) and follow-up (Peitzmeier et al. 2014) compared to cisgender patients.

Various case studies in the health literature point to the cancer risk profiles of trans and/or gender nonconforming people and the potential vulnerabilities and/or protective factors that are associated with medical interventions such as hormones or surgery (Bentz et al. 2010; Pattison and McLaren 2013). This is important in that sexual and gender minority populations are frequently overlooked in research on cancer health disparities (Gibson et al. 2017; Sinding, Barnoff and Grassau 2004). Significantly fewer studies have focused on QLB experiences of gynecologic cancer than of breast cancer, and our project addresses this gap directly. Our literature review located virtually no research published concerning gender variance and experiences of breast or gynecologic cancer health and treatment decision-making (exceptions include: Bryson and Stacey 2013; Margolies and Brown 2018; Taylor and Bryson 2016). Efforts to understand and facilitate knowledge mobilization among health professionals and QLB women and trans people require attention to how gender and sexuality operate intersectionally and relationally.

### Intersectional complexity and “women’s cancers”

Cancer health research that has included to-date a focus on minority sexuality has tended overwhelmingly to include only “lesbians” as if constitutive of, or equivalent to, a homogenous, coherent and ahistorical whole while persistently overlooking the significance of dynamic gender differences *amongst* QLB breast and gynecologic cancer patients and trans patients (Burkhalter et al. 2016; Fentiman 2017). The purposive and systematic attention to gender, as *intersectionally linked* with sexuality relative to experiences of accessing and mobilizing cancer health and treatment knowledge, constitutes one of the significant advances the *Cancer’s Margins* project makes with respect to research on breast and gynecologic cancer classified under the rubric of “women’s cancer” health and care (Crenshaw 1991; McCall 2005).

There is a sizeable body of knowledge identifying both sex and gender as key determinants of health, including cancer health, that index differences not attributable uniquely to biological sex effects (Moynihan 2002). Building on literature that has identified a unique relationship between cancer, minority sexuality and gender identity, expression, and embodiment (Jain 2007a) as well as health disparities and precarities (Feinberg 2001; Jain 2007b), our research critically interrogates binary models of sex/gender reified in the biomedical classification of breast cancer and gynecologic cancers (cervical, ovarian, uterine, vaginal and vulvar) as “women’s cancers” (King 2006; Klawiter 2004). Research provides robust evidence that gender expression differences *amongst* QLB women (e.g., butch/femme continuum) intersect with sexuality, which together impact health behaviors, vulnerabilities, experiences and beliefs (Hiestand, Horne and Levitt, 2007; Rosario, Scrimshaw and Hunter 2008).

*Cancer’s Margins* (www.lgbtcancer.ca) is likely the first nationwide study to investigate the complex intersections of sexual and/or gender marginality, cancer knowledge, treatment experiences, and modes of organization of support networks. We address three interconnected gaps in the current research evidence. First, the limited research regarding sexual and gender minority populations and breast and gynecologic cancer has focused on between-group differences in health beliefs, behaviors and outcomes, that is, participation in cancer screening, or satisfaction with healthcare providers. Whereas quantitative evidence of cancer-related health disparities indicates the sheer existence of inequalities, there is urgent need for explanatory, qualitative accounts of experiences and attention to narratives of cancer health and care decision-making linked with these inequalities. Second, it has been common for sex and gender to be conflated in biomedical (and quotidian) systems of cancer care, and for gender to be treated as a stand-alone “social determinant” of health without any recognition of its intersections (Kazanjian and Hankivsky 2008; Numer and Gahagan 2009). And so, it remains common practice for breast and gynecologic cancers to be classified as “women’s cancers,” resulting in neglect of multidimensional within-group differences. Third, research on sexual minority populations’ experiences with breast and gynecologic cancers has overwhelmingly focused on lesbians, and has neglected the significance of sexual diversity, along with gender diversity, and the dynamic, generational elements of sexual and gender identification across time and place (Kerker, Mostashari and Thorpe 2006).

## Theory and method

*Cancer’s Margins* adopts the general theoretical orientation known as the Social Study of Medicine (SSM): an empirico-theoretical, sociocultural approach to the study of health as coextensive with (a) the study of knowledge, and (b) the treatment of knowledge and its uneven mobilities within and across divides as located within complex biopolitical systems of knowledge (Diedrich 2007; Mol 2002; Patton 2007; Spurlin 2018). We enlist narrative methods so as to treat knowledge archeo-genealogically; that is, as multiple, complex and embedded in knowledge ecologies that constitute local “assemblages of intelligibility” over time and across communities. We ask, *what* and *who* counts as knowledge and knowledgeable, and as worthy of locating, remembering, unsettling and/or contributing to, utilizing and sharing. This research is productively located at the nexus of two social science trajectories that have, historically, been considered incommensurable. On the one hand, *genealogical articulations* of “systems” or “fields” of knowledge (often flagged as Foucauldian) and, on the other hand, *phenomenological accounts* of embodied experience as “lived experience.”

The *Cancer’s Margins* study uses an intersectional and relational approach to cancer, sexuality, and gender (Johnson and Repta 2012; McCall 2005; Meyer 2012). We focus analytically on cancer patient narratives to trace complex experiences of health, illness, and the challenges to a stable sense of embodiment that inevitably arrive in cancer’s wake. Our analysis focuses on patient narratives as autobiographical artifacts makes use of the concept of “choreography” as providing both a lens and a method with which to appreciate intense local modes of epistemic and ontological dissonance and alignment that arise in the cancer clinic, between multiple actors, and multiple modes of biographical and biomedical cancer knowledge, across the trajectory of cancer health decision-making. Charis Thompson deployed the concept of “ontological choreography” (2005) as an analytic tool and as a descriptor of the dynamic processes of objectification, agency and subjectification observed in infertility clinics. There are similar dynamics at play in the cancer clinic. Cancer patients generally, but in particular, LGBQ//T2 folks, encounter biomedical knowledge in multiple guises (care provider, website, pamphlet) that objectifies the cancer patient, organizes them into a modality suitable for a lock-step trajectory of treatment protocols, and that typically renders invisible or unacceptable, non-normative biographical knowledge about bodies that are differently gendered or sexualized (Guthrie 2018; Jain 2007a; Joynt and Bryson 2012). The idiom of choreography provides for a mode of analysis that is attentive to the simultaneous coordination of bodies, knowledge and relations of power and also, the inevitably dynamic quality of queer and trans bodies that refuse to sit still, and that insist on modes of resistance which, in the face of cancer, re-write canonical cancer knowledge and sometimes refuse cancer treatments intended to reproduce embodied normalcy (Bryson and Stacey 2013).

Purposive sampling methods were utilized to recruit a diverse sample of participants diagnosed and treated for breast and/or gynecologic cancer in relation to: age, race and ethnicity, stage of cancer diagnosis, type of cancer, socio-economic status, dis/ability, gender identity and expression, and sexual identity (Bauer et al. 2017). Participants were recruited from urban, suburban, and rural locations in the Canadian provinces of British Columbia (BC), Manitoba (MB), Ontario (ON), Quebec (QC), and Nova Scotia (NS). The sample (see Table 1) includes pilot interviews with LBQ//T2 people diagnosed and treated for breast and gynecologic cancer living in the San Francisco Bay Area (BA). Research ethics approval was sought and granted by each of the investigators’ Ethics Review Boards.

**Table 1:**
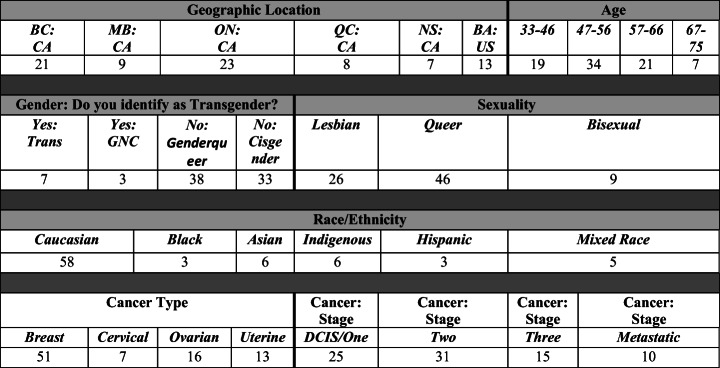
Cancer’s Margins Demographics (*n*=81)

Semi-structured interviews were carried out with 81 participants (see Table 1) who were assigned female at birth, and who self-identified as queer, lesbian or bisexual and/or as transgender (includes gender nonbinary or gender nonconforming) or Two-Spirit and who had been diagnosed and treated for breast and/or gynecologic cancer within the past five years. It is notable that, in response to questions about gender identity and expression, 38 of the participants who did not self-identify as transgender (71/81) nonetheless provided a highly diverse set of genderqueer identity terms and 31 self-identified as “cisgender” or “woman”. The interviews were conducted in French or English. Participants ranged in age from 33-75 years of age. The interview protocol utilized for the project elicited life story narratives, and in particular, narrative accounts of diagnosis and treatment. Prompts followed a cancer trajectory that typically includes: pre-diagnostic participation in screening, cancer diagnosis, surgery, and subsequent treatments (i.e. chemotherapy, radiation, hormone therapy) and follow up care, surveillance and/or metastatic care. Interviews were focused on the experiences of cancer and cancer treatment for sexual and/or gender minority people, participants’ organization of cancer and health knowledge, knowledge access and mobility, cancer and health decision-making, and access to support networks. Interviews were about 120 min in length and conducted by interviewers familiar with the complexities and vulnerabilities of sexual and/or gender minority people diagnosed and treated for cancer.

Qualitative data analysis was carried out in multiple stages, including: (1) development of initial codes: biographical self-knowledge, biomedical cancer health and treatment knowledge, mobilization of knowledge in decision-making and designs for support during and after treatment; (2) refinement of coding system to include 8 major coding categories and 63 sub-codes; (3) training and inter-rater reliability assessments of the complete coding system and 30% of all transcripts to acceptable levels of agreement (80% and higher); (4) coding of all 81 transcripts using MAXQDA qualitative research software. Our subsequent analysis of interview data was based on searches of the transcripts designed so as to inquire into the coordination of systems of biographical and biomedical cancer knowledge, and knowledge access and mobilization practices pertinent to cancer health decision-making among LGBQ//T2 breast and/or gynecologic cancer patients from varied social and cultural contexts.

## Analysis and discussion

Our research investigates how gender and sexuality are intersectionally salient to sexual and gender minority cancer patients’ experiences of cancer care, health outcomes, and decision-making across the “cancer trajectory” (primary prevention/screening, during and after cancer diagnosis, surgery and adjuvant treatments, and survivorship). In addition, we examine how access to culturally relevant breast and gynecologic cancer knowledge is negotiated and how incommensurabilities in knowledge and related communication difficulties, are navigated. In particular, we draw on the use by health researchers of “*choreography*” as an idiom that aptly characterizes the dynamic scene of healthcare decision-making for minority cancer patients (Thompson 2005; Vayrynen et al. 2017). In the fraught mix of LGBQ//T2 cancer patients and the cancer clinic is an assemblage of multiple systems of knowledge, actors and practices that co-exist in health settings that are characterized by robust and persistent differences in power, and yet, that also provide anecdotal evidence of the multiple forms of agency that exist in encounters between bodies located in state systems. Our objective is to document how patients design, experience, resist, and navigate improvisatorially, complex choreographies of access to, and negotiation of, biographical and biomedical knowledge related to cancer health and treatment decision-making. As we noted elsewhere, “Improvisatory performances of being with cancer and of feeling cancer seem closely attuned to the kinds of queer practices of freedom, and relatedly, the modes of unanticipated local mobility, that such an explicitly politicized, collective and agonistic engagement with cancer’s inevitable alterity occasions” Bryson and Stacey (2013, 206).

### Awkward choreographies: disclosures about sexual and gender identity in care settings

Minority stress and the social stigma linked with both sexual and/or gender minority cancer patients’ histories of discrimination and relatedly, “institutional and informational erasure” seep into and in many cases, underwrite experiences of cancer care communications and contexts. There is a strong relationship between perceptions of cultural safety and experiences of sexual and/or gender minority cancer patients. In particular, an obdurate element of cancer care for sexual and/or gender minority patients is a perception of an intrusive level of risk linked with disclosures of sexual and/or gender minority identity in cancer care environments.

*“I was really worried about how I’d be treated as a dyke and how my partner would be treated. There was only one care provider, which was a nurse, when I was in the Recovery Room. That nurse was obviously really not okay with me being a lesbian, or trans, or whatever. She was not okay. Fortunately, she went off shift before I left Recovery and went upstairs to be hospitalized.”* **Jolene** (ON, 59, Caucasian, genderfluid, queer, breast cancer^2^)

“*When you get diagnosed, the whole process feels like such a steamroller. What I had felt previously in my life was if I want people to know I’m bi and queer, I have to out myself pretty much every time. God. Try that in the medical system. You know. Suddenly it goes from difficult to just irrelevant. I had all these moments in treatment where I was like, ‘Do I say something? Do I say, “By the way I’m bisexual.”? … Medicalization can be erasing of so many aspects of your identity. I felt completely invisible. It was awful.”* **Serena** (BC, 39, Caucasian punk femme bisexual, breast cancer)

For many of the racialized *Cancer’s Margins* participants, identification as a sexual and/or gender minority person and related issues of minority stress included a heightened awareness of the impacts of racialized cultural knowledge and of racism to produce intersectional forms of both LGBQ//T2 self-knowledge as well as additional layers of risk in institutional settings of care.

“*When I was first coming out, my girlfriend was white. And she did women’s studies courses. (laugh) There was one chapter in her Women’s Studies book on Black Feminist or Black Lesbianism or something. I read that. And then the bibliography. Then I went in search of these people. It was so amazing. As ludicrous as this sounds, I thought I was the only one. … I was living in a very small Ontario town. … A lot of my experiences weren’t positive. Of racism. … People yelling at me from cars… People following me around stores. Of some facility not giving me the benefit of the doubt in things.”* **Florence** (ON, 41, Black Caribbean, lesbian woman, breast cancer)

Cisgender sexual minority women typically reported simultaneously that while they weren’t discriminated against *directly*, that nonetheless, they were unsatisfied or had negative interactions with cancer care providers. The *Cancer’s Margins* LGBQ interviewees who had experiences of discrimination typically described that their cancer care provider was visibly and tangibly uncomfortable in their presence. As Louise was making pain management plans and end-of-life decisions, she observed both homophobic and racist attitudes on behalf of the palliative care provider.

“*I was referred to Palliative Care in the community. It’s in a Catholic hospital. I went in to meet with the Palliative Care doc. Her demeanor and the way she spoke with me, and how she responded was bizarre. I was asserting what was important to me in terms of discussions with my partner and around my care at home. And she said, ‘Oh. You don’t need to worry about that because when the time comes, I will assess how you’re doing. I will tell your, ah, you know, tell your family.’ It was really clear to me that in terms of homophobia, and also in terms of racism, I really had absolutely no confidence that she would be somebody who myself or Surita would be comfortable with. I just didn’t trust her. The discomfort was palpable. It was a horrific experience. I walked out of there thinking, ‘Oh my god. That was homophobia. That’s what that was.’”* **Louise** (ON, 46, Caucasian, Femme, lesbian, queer woman, ovarian cancer)

Many transgender and/or gender nonconforming patients reported experiencing various forms of service refusal and denial of care or support.

*“I showed up for my appointment and I was immediately told, ‘You are in the wrong place, sir.’”* **James** (BC, 58, Caucasian, trans man, queer, cervical cancer)

*Cancer’s Margins* interviewees canonically made decisions about disclosing sexual or gender identity matters after evaluating issues of safety and the possible impact of disclosure on quality of cancer care. Interviewees related that in so doing, they found themselves navigating an awkward choreography of ill-fitting practises between sharing with care providers what matters about themselves and responding to forms and patient-provider scripts that seemed to accord no place for patient biographical knowledge about sexual or gender identity.

### Fraught choreographies of care: treatment decision-making and incommensurabilities in communication with providers

Interviewees prioritized gender, parenting and fertility, sexuality, and other aspects of intersectional identities and complex, multi-faceted queer and trans lives in their cancer treatment decision-making. Most folks reported accounts of treatment interactions replete with significant communication difficulties with care providers. Providers showed little evidence of struggling to understand (and accept) cancer patients’ decision-making when it contravened normative expectations of identity or of embodiment. *Cancer’s Margins* interviewees related that it appeared as if the fact that on the whole, breast and gynecologic cancers are biomedically and culturally coded as “women’s cancers”, impeded their access to culturally competent care. Many of our interviewees were confident that they were not part of the group of “women” that biomedical discourses concerning “women’s cancers” imagined and hailed to congregate under an umbrella replete with pink ribbons (King 2006).

Ariel described communicating to a cancer provider that their decision to undergo a bilateral mastectomy was informed by their experience of seeing others in their community undergo mastectomy (top surgery) in the context of gender affirming care. In Ariel’s story, a male oncology surgeon seemed both unfamiliar and uncomfortable with this decision and sent them off to see a female cancer care provider, which resulted in multiple delays in care – a denial of care that may be directly related to the cisnormative knowledge that informs systems of cancer care.

*“So, he started in with, ‘We could do a lumpectomy.’ and ‘Blah, blah, blah.’ I think even the first night I had already decided I wanted a bilateral mastectomy. It was really immediate and strong for me. So I said that. And he’s like, ‘Why would you do that?’ And I’m like, ‘There's a lot of women in my community who are transitioning. You know - having top surgery. I don’t see this as a bad option.’ The way (my partner) blogged about it was, ‘You could just see his little mind peddling as fast as it could to try to keep up with what we were talking about. ‘Cause he had no frigging idea.’ I think we really unnerved him. Then we went to go see another surgeon, and it was the same thing.”* **Ariel** (BA, 50, Latinx, genderqueer, queer, breast cancer)

Liwayway described an interaction with a gynecologic oncology care provider who struggled not only to understand their decision-making process and consideration of a prophylactic mastectomy while negotiating treatment for ovarian cancer, but who also completely misunderstood how the administration of hormones pertinent to their gender affirming care ought to be coordinated with their cancer care. This provider accused Liwayway of “trying to get free surgery.” The lack of the provider’s cultural competency regarding gender affirming care practices negatively impacted the provision of medically appropriate and culturally safe/r ovarian cancer care for Liwayway.

*“I help a lot of young transgender men. At that time, they thought that my cancer had spread to my breasts. I talked to my doctor. ‘If it's spread to my breasts, take them off. Mastectomy.’ I talked to the trans men, to see which doctor they go to to have their breasts removed. I said, ‘I got cancer and there's a possibility my cancer will spread to my breasts.’… Why would I put silicone in my boobs? To cause another cancer? And besides, you know, I'm a drag king. I play a lot of roles being a man. So I decided to be flat chested. The doctor was accusing me of trying to get free surgery to get my breasts removed. Even though I have cancer.”* **Liwayway** (BA, 50, Filipino/Aboriginal, genderqueer & trans, Two-Spirit, ovarian cancer)

Interviewees reported gendered aspects of embodiment as extremely important to wellbeing after treatment. Following top surgery carried out as part of his gender affirming care, John was treated for breast cancer. John reported making special efforts to have his breast surgeon understand his wish preserve the aesthetic results achieved by the top surgery. John’s wellbeing as a trans man was partly based on his ability to present an embodiment consistent with his gender.

*“It was very soon after my chest surgery that I found the lump. The cosmetic surgeon said, ‘If you can tactfully do it, here’s what I would tell the surgeon to do to minimize the scarring.’ It was a bit of vanity. I think body image is a really difficult thing for a lot of people. And particularly in trans people. I gave the surgeon instructions. I'm like, ‘Can you please cut along the scar if you can?’ He did try. But it does look a little different. With the radiation, the scars are a different colour. They didn’t heal as well as the other side. With being off Testosterone and being on Tamoxifen everything is a little puffier. It still looks good in clothes, which is important. Keeping my nipples was important to me to maintain a chest that I would be able to go shirtless with in the not too distant future.”* **John** (ON, 33, Chinese-Canadian, trans man, queer, breast cancer)

When cancer care providers’ communication with patients doesn’t address or in any significant way take up proactively, the nuanced and important relationship to biographical self-knowledge concerning identity or embodiment for sexual and gender minority cancer patients, a significant aspect of the actual knowledge that is informing patient decision-making goes unaddressed. Despite being located in healthcare institutions that stipulate patients’ rights to both medically and culturally competent cancer care, it would appear that few cancer providers are informed about LGBQ//T2 gender and sexual identities and their relevance to the design of evidence-based appropriate care.

### Generational differences in narratives about LGBQ//T2 identities and health knowledge

*Cancer’s Margins* participants from distinct generational cohorts related biographical narratives about sexual and gender identity, and approaches to health information seeking that were located in distinct generationally bounded knowledge regimes.

LGBQ participants 33 to 56 years-of-age typically related narratives about the articulation of a queer self and community where a great deal of formative activity took place in the late ‘80’s and ‘90’s. These participants were more likely to identify as “queer” than as “lesbian,” to recount a socially constructed and non-reductive gender identity that is differentiated from biological sex alone, and to seek information from medical providers or online. Younger participants were more likely to presume that care providers should be attentive to and aware of their needs as members of a marginalized population(s).

LGBQ participants 57-75 years-of-age “coming of age” stories about sexuality were primarily located in the late 70’s to mid-80’s. These interviewees were more likely to identify as “lesbian” and “woman.” They tended to view health knowledge access within an “*Our Bodies Ourselves*” lens, which is to say, as distributed across peer-to-peer community relationships and as actualized by means of a “DIY” lesbian politics of health and wellbeing (The Boston Women’s Health Book Collective 1973). These participants were more ardent in their choices about preferring female health providers. They tended to report a more distrustful relationship with health and care providers and relatedly a health narrative that contextualizes “women’s health” as a political, feminist issue where community relationships provide access to culturally appropriate and trustworthy health knowledge. Cheryl (BC, 65, Caucasian, woman, dyke/lesbian, uterine cancer) recounts that immediately after hearing the diagnosis of uterine cancer from her GP, that to inform her forthcoming conversation with a gynecologist about surgical decision-making she, “*called up every woman I knew who’d had a hysterectomy. We didn’t get a second opinion, we got dozens*!”.

All participants who identified as transgender described experiences of accessing support, knowledge and in some cases, gender affirming medical care, that took place over the past decade (and not prior to that time), which is consistent with the timeline of when knowledge, support and care regarding gender and transition for people whose gender assigned at birth was female became somewhat available in North America.

*“That’s how I identify. Over time, I have added the gender queer to the queer. That's been a long time coming. But definitely with the cancer diagnosis… With the bilateral mastectomy… With the more trans affirmative kind of community that I belong to now… It feels like that really opened up a comfortable space for me, and my body, and so forth.”*

**Ariel** (BA, 50, Latinx, genderqueer, queer, breast cancer)

The significance of bringing attention to “generational cohort” as an important element in the multiple and dynamic field of LGBQ//T2 identities is that it provides an interesting place where we can see the value of an intersectional analysis that complicates simplistic or essentialist notions about monolithic, or seemingly ahistorical “LGBT” cancer stories.

### Gender “in treatment”: cancer as site for resistance, repair and the disruption and reworking of major biographical narratives

Cancer treatments invariably include surgery as a key event in the trajectory of patient and provider decision-making and experiences of treatment. In particular, breast and gynecologic cancer surgeries (e.g., mastectomy, hysterectomy) present decision-making challenges to cancer patients that include significant threats, risks and disruptions to a person’s felt sense of gender and sexuality. LGBQ//T2 narratives concerning interactions with breast cancer surgeons provide detailed testimonials concerning the various incommensurabilities between biomedical and biographical discourse about cancer. Most *Cancer’s Margins* interviewees who talked about surgical decision-making in breast cancer treatment that included mastectomy as one of, or the only, surgical option, reported that breast reconstruction was framed by surgeons in canonical treatment decision-making scripts as a necessary, inevitable, beneficial and normal sequel to mastectomy. As Manon (MB, 41, French Canadian lesbian, breast cancer) reported, “*I went and saw the oncology surgeon who does the mastectomy. The very first thing she said right away was, “Well. Who do you want to have to do the reconstruction?”*.

The preponderance of LGBQ *Cancer’s Margins* interviewees facing decision-making about breast cancer surgery elected bilateral mastectomy without reconstruction or lumpectomy. Patient narratives make it abundantly clear that in almost all of these cases, cancer surgeons were completely unaware and disengaged from, and in some cases, resistant to, the actual significance of patients’ felt sense of gender as a critical knowledge source. For many *Cancer’s Margins* interviewees, a felt sense of gender informed their resistance to their surgeons’ typical approach to the biomedical presentation of the apparent inevitability of breast reconstruction. Biographical knowledge served as a means to retain and repair disruptions to a sense of phenomenological coherence occasioned by cancer surgery. Jolene reported how, in spite of the fact that their life story has included a significant and persistent array of forms of resistance to cisnormative gender identity and a great deal of fluidity in relation to modes and styles of approximating kinds of female masculinity, that it is only in the face of breast cancer that they became aware that the resulting change in embodiment provided by mastectomy could actually be reparative of what they had experienced as a failure to reconcile embodiment and gender identity. Jolene’s report of various communication breakdowns in their interaction with the breast cancer surgeon is typical of many such accounts that we have witnessed in this project, of incommensurabilities and extraordinary efforts on the part of patients to advocate for their own decisions about surgeries.

*“I knew I had cancer. I knew I had to have an operation. I knew that I wanted a double mastectomy. There was no question. The first surgeon was really uncomfortable with me having a double mastectomy. I had to push. People kept asking me, ‘Are you sure you don’t want implants?’. I had to go and get a second opinion for the mastectomy.”* **Jolene** (ON, 59, Caucasian, genderfluid, queer, breast cancer)

Most interviewees improvisatorially sketched hybrid biomedical and biographical narratives to make sense of cancer surgeries. For many LGBQ interviewees, it was very clear that cancer patients invoked community-based knowledge of gender fluidity and transgender identity and expression to produce alternative and counter-normative narratives about post-surgical bodies. Whereas typical biomedical narratives about the impacts of cancer surgeries make extensive use of the tropes of surgery as a ‘return to normalcy’ or a ‘body that is again made whole’, many Cancer’s Margins interviewees deployed queer and trans narratives that provided them opportunities for agency in electing other outcomes, like “going flat” after mastectomy. Holly described how her bilateral mastectomy (without reconstruction) impacted her sense of herself as a bisexual person who identifies as ‘queer’ and as ‘femme’. For Holly, mastectomy affected her gender expression in the direct sense that she elects sometimes to wear a prosthesis, and the rest of the time to not wear a prosthesis – a state of embodiment she describes as “being flat”. Holly invoked transgender narratives of gender embodiment as providing her with forms of discourse and awareness that were available to her in particular because of the queer geographies of sexuality and gender in the San Francisco Bay area.

*“Having breast cancer … I’ve had almost all of my biological female parts removed. I’m just at the beginning of thinking about what that means in terms of my identification as female. I sent out this wry email to my friends who get this stuff, saying, ‘Thank god gender is a social construct or I wouldn't be a woman anymore.’ You know? I am just trying to deal with my anger of having these precious parts of me removed. I wrote about this in the blog, early on. (sigh) I'm so lucky to be living in an area where some people choose not to have breasts and celebrate that. I can learn from them, right? I don't know what that will do to my identity in the long run.”* **Holly** (BA, 44, Jewish, femme, queer bisexual, breast cancer, BRCA1)

A significant subset of the *Cancer’s Margins* interviewees who identified as “femme” and who were less than 50 years-of-age at diagnosis elected reconstruction. Interviewees who elected reconstruction reported the prioritization of normative values concerning breasts, body image and femininity and/or an unwillingness to question oncology surgeons’ narratives linking mastectomy and reconstruction as if a single and inevitable sequence of surgeries. Serena was diagnosed with the BRCA2 gene mutation, which carries an elevated risk of both breast and gynecological cancers. Prior to her own breast cancer diagnosis, Serena did extensive research online concerning prophylactic mastectomy and oophorectomy, due to her sister’s prior diagnosis of BRCA2. Prophylactic mastectomy with reconstruction was a plausible option for her own proactive approach to her higher breast cancer risk.

“*I was on all the chat boards, trying to read the threads of who had chosen to get prophylactic mastectomy and why, and what were the consequences and did they get reconstruction.”*

**Serena** (BC, 39, Caucasian, punk femme, bisexual, breast cancer)

For several participants diagnosed at an atypically young age with breast cancer, it was clear that electing to undergo reconstruction at the time of the bilateral mastectomy was a very significant way to preserve what mattered to them about the significance of breasts to their gender identity and expression as femmes.

“*I accept the trappings of femininity. … Make-up, clothes, all those sorts of things. … I would say, ‘Definitely femme, with a punk edge.’. I’m a punk femme.* … *I felt and still feel fairly strong about reconstruction. My mother chose not to get reconstruction. Everybody makes it their own choice. I immediately felt that I needed to do reconstruction, as did my sister. … I had a conflation in my head of reconstruction and augmentation. I was trying to be playful with the whole thing. Maybe this can be okay. Maybe this can even have some nice benefits. I looked online at pictures of reconstruction. I am deeply grateful that there are pictures online of reconstruction.* **Serena** (BC, 39, Caucasian, punk femme, bisexual, breast cancer, BRCA2)

“*If I was BRCA positive, I would have gotten a double mastectomy and then implants. But when it came back negative, I decided to get a lumpectomy. … I like my breasts. I’d like to keep my breasts. I feel like they are part of my sexuality. I would feel very sad (voice breaks) if disease was present and I had to get a double mastectomy*.” **Terry** (BA, 40, Caucasian, femme, queer, breast cancer)

For some sexual minority interviewees who identified as gender nonconforming but not transgender, the fact that mastectomy and hysterectomy for breast and gynecological cancer are essentially the very same procedures undertaken by some transgender folks as part of gender affirming care created a unique barrier to the singular prioritization of wellbeing. The likelihood of being misidentified as transgender following mastectomy carried out as part of breast cancer treatment was, for some participants, an unacceptable outcome. This overlap in the significance of gender affirming and cancer surgical procedures appears to be completely unknown to health care providers and yet was discussed by many *Cancer’s Margins* interviewees. For Jake, the added likelihood of being misrecognized as a trans guy meant that it was simply unacceptable to address the higher probability of breast cancer linked with the BRCA1 gene mutation by electing to undergo a prophylactic mastectomy following treatment for ovarian cancer.

“*They told me I have an eighty percent chance of getting breast cancer in my life. … The oncologist is always going on about doing a prophylactic mastectomy. … Because I don’t ID as a trans guy, I feel like I would almost become one by default, if I had a mastectomy. I feel like if I do that I will have bought into a binary gender system. … It’s not like I have a great big rack. And it’s not like I feel super attached to them. It just comes down to some kind of gender identity thing. I don’t feel like the word woman fits on me.”* **Jake** (BC, 52, Caucasian, butch dyke, ovarian cancer, BRCA1)

For those *Cancer’s Margins* interviewees who identified both as transgender and as sexual minority breast or gynecologic cancer patients, it was very clear that the overlap in gender affirming surgeries (e.g., top surgery or hysterectomy) and cancer treatment surgical procedures created the basis for a wholly differentiated form of gender-based cancer health decision-making. Surgical decision-making about cancer treatment was underwritten by a commitment to the belief that the impact of the surgical procedure on gender identity and expression was unilaterally positive and quite simply a means to receive gender affirming treatment secured by means of cancer treatment. Blake was already scheduled for top surgery when he was diagnosed with ovarian cancer. Blake’s narrative about his conversation with the oncology surgeon indicates that he was responding to the information about the forthcoming ovarian cancer treatment surgery as if it were actually surgery being carried out as part of a plan for gender affirming care.

“*I saw the oncology surgeon on February 9*^*th*^*. She said, ‘We’re going to do a hysterectomy. There’s a chance that it will be cancer. There’s a chance that it'll just be cysts. It’s a large complex mass.’ So I said, ‘This is good news. I wanted a hysterectomy anyway. I’m going to get this part done first.’ Works for me. To me, it was all positive. So, in that sense, although I was told, ‘There’s a possibility of cancer here.’ that’s not what I heard. What I heard was, ‘I get a free hysterectomy.”*’ **Blake** (BC, 57, Caucasian, trans man, ovarian cancer)

Whereas previous research has suggested “wellbeing” as a priority in sexual minority women’s cancer decision-making our findings contribute several additional and more nuanced perspectives. Paying attention to intersectional specificities in cancer patient narratives has allowed us to identify several distinct biographical modes of self-knowledge that play a major role in how LGBQ//T2 cancer patients experience and resist biomedical modes of cancer treatment decision-making. Interviewees deployed queer and trans narratives concerning gender and embodiments to make sense of their cancer treatment process, to support them in biographical repair, and to inform treatment decision-making. Knowledge of various trans narratives gives sexual and gender minority breast and gynecologic cancer patients an alternate knowledge system to the biomedical scripts presented to them by cancer care providers across a highly choreographed cancer treatment trajectory. Interviews provided a great deal of evidence that in the face of decision-making related to cancer surgeries, many LGBQ and Trans cancer patients resisted cisnormative biomedical cancer narratives and in their place, created complex and fluid biographical stories which served a key role in permitting a kind of biographical repair carried out by means of the deployment of non-normative narratives concerning changes to gendered embodiment; narratives quite distinct from those cisgender narratives of breast and gynecologic cancers reified as “women’s cancers” alone.

### Cancer-related treatments impact felt sense of gender

While there is no published research on treatment side-effects for gender minority populations, our findings suggest cancer treatment can change aspects of embodiment in such a way that creates experiences of gender marginality for sexual minority women who otherwise identify as cisgender women. These participants experienced changes to their embodiment that shifted their relational gendered self-knowledge and expression. The changes to gendered embodiment made sexual minority cisgender breast and/or gynecologic cancer patients acutely aware of the ways in which their access to public space, knowledge, or mobility was affected by these bodily changes.

Charmaine’s hair didn’t entirely grow back after chemotherapy treatment, and so she was left with a receding hairline that resulted in being perceived as male in public. Charmaine struggled to figure out how to alter her gender expression to simply use a public washroom.

*“No, I don’t consider myself to be trans or gender nonconforming. But my hair is stupid post-chemo, so I’m just mistaken for a man a whole lot… I don’t want to do a comb-over. I don’t want to freak people out. What happens is that I walk into the women's washroom and women scream. You know? And I’m just…. ‘Really, I just have to go to the washroom, ladies.’ (laugh). The male pattern balding is easily misread as male.”* **Charmaine** (MB, 51, Caucasian, female, dyke/lesbian, breast cancer)

Like Charmaine, Olivia’s embodied experience of gender and cancer treatment intersect and create an incongruence between normative embodiments of gender and her experience of gender expression as it intersects with her post-treatment embodiment. While Oliva feels like a woman, she suspects that her embodiment is not read as female. This incongruence is identified as a marked form of gender incoherence that she understands through the knowledge system of a “trans” location in relation to gendered embodiment.

*“When I’m walking around on the street and I meet other people, I’m not sure I’m being perceived as a woman.* P*eople must say to themselves, ‘Is this a woman, a man, a boy, or an old man?’. When I look at myself in the mirror, I ask myself, ‘Who am I?’, ‘Am I still a woman?’. And the answer is ‘Yes.’. I am still a woman for my partner. And for the friends who know me… I am a woman. For society, I may be more of a trans person. I understand my road. It’s a road that includes removal of my breasts. I don’t feel like putting on a prosthesis and then a bra. It’s so uncomfortable. I don’t want that… I will probably develop new behaviours to facilitate interactions with people.”* **Olivia** (QC, 60, Caucasian, lesbian woman, breast cancer)

Communication between sexual and gender minority patients and their care providers is fraught and this negatively affects patients’ experiences of care**.** The lack of availability of inclusive cancer support groups for sexual and gender minority cancer patients was discussed by many as one major element that intensifies the detrimental effects of LGBQ//T2 folks’ lack of access to knowledge resulting from impoverished communication with providers.

For instance, participants reported that providers did not prepare them to make informed decisions or to cope with the changes in embodiment and endocrine systems that would result from hormone treatments and/or surgical menopause. Jake talks about how she wasn’t prepared for the changes in her affect as a result of her surgery for ovarian cancer.

*“It’s like, ‘So we cut out all these parts. Cancer, cancer, cancer.’ Nobody mentioned surgical menopause. I just remember sitting in Trout Lake, with (ex-partner) and her saying, ‘Why are you crying?’ And me saying, ‘I don't know.’ But I couldn't stop crying. I’ve never felt so out of control in my life. Having my head shaved and then having these hot flashes come over me. My entire scalp would be soaked in sweat, in a minute. I remember thinking, ‘There’s no such thing as mental illness. It’s all hormones.’ Because I was completely nuts. I was completely unprepared for it.”* **Jake** (BC, 52, Caucasian, butch dyke, ovarian cancer, BRCA1)

Sibyl also describes not being provided the option by her providers to discuss the administration of hormones after cancer surgery.

*“The Sexual Health doctor was there at the presentation. I went up, because she was talking about the regime that they do. How you are put on hormones right away while in the hospital and, then you're tapered off in a year or two. And I'm like, ‘That never happened for me.’ So, I actually went up to her after, and I said, ‘Was there a reason? This is my story.’ And she said, ‘No.’ She said, ‘The boys dropped the ball.’ She said, ‘You should have had hormones.’ I was slammed right into menopause. I didn’t know what was brought on by surgery and what was from the chemo.”* **Sibyl** (BC, 54, Caucasian, gender fluid, lesbian, ovarian cancer)

The lack of clear communication from provider to patient, particularly around sexual health and menopause in the face of cancer treatment, has also been well-documented in the larger cancer literature (e.g., Charif et al. 2015; Scanlon et al. 2012). However, since culturally competent modes of support frequently do not exist for sexual and gender minority patients, the impact of disparities in care worsens already existing population differences.

### Support networks

*Cancer’s Margins* cisgender LGBQ participants relied more on partners and friends for support than biological family, as compared with evidence from heterosexual women diagnosed with cancer. Strained social relations as a result of homophobia and heterosexism contribute to social isolation and fragile care networks. Our findings support the claim that gender diverse populations face the effects of compounding marginalization that contributes to social isolation and barriers to accessing support, particularly cancer support groups.

“*I went to one of the Wellspring support groups... I just went once and it wasn’t for me. I wish there was a therapy group dealing specifically with the gay and lesbian community. Heterosexuals’ experiences are different. When you’re sitting in a group that’s all heterosexuals and they’re talking about their kids, the husband and you know – blah, blah, blah – I didn’t find those groups fulfilling at all.”* **Leah** (ON, 51, Black, soft butch, lesbian, breast cancer)

Interviewees reported difficulties in accessing provincial cancer peer support programs.

“*I said, ‘Can you do a key word search for FTM or trans? Anybody trans?’. They’re like, ‘No, we can’t.’ Nothing came up. They’re like, ‘Do you want a lesbian or do you want a gay?’ It’s like, ‘I don’t want to talk to a lesbian. That’s a different thing. And I don’t want to talk to a gay man.’ And so they’re like, ‘Well. Do you want a man or a woman?’ You have to choose all those things where none of them fit. It was so uncomfortable. I ended up saying, ‘Okay. Have a man call me.’ They had a man with breast cancer on their support list. He couldn’t really answer my questions.”* **John** (BC, 33, Chinese-Canadian, trans man, queer, breast cancer)

Cisnormative knowledge systems negatively impact and shape systems of care, which create additional and specific vulnerabilities for gender diverse patients related to accessing support during treatment. Trans and/or genderqueer participants reported additional challenges to accessing care and support during treatment and had less family/friend support compared with cisgender LGBQ participants. In fact, some trans participants clearly stated that their family members created stress during treatment and that this additional stress seriously impacted their cancer treatment decision-making.

## Conclusions

*Cancer’s Margins* was designed to advance our understanding of how sexuality and gender are intersectionally constitutive of complex biopolitical networks of cancer health knowledge that shape knowledge access and its mobilization in health and treatment decision-making. As we noted in an earlier article from *Cancer’s Margins*, “Social and cultural research on health experiences brings critical attention to the demands that illness places on people who become “patients,” to choreograph access to knowledge and care across significantly distinct and sometimes incommensurable systems of knowledge including “lay understandings” of self and health versus biomedical explanatory knowledge systems” (Bryson and Stacey 2013, 204).

The LGBQ//T2 cancer patient narratives we have analyzed here document in fine grain detail how it is that sexual and/or gender minority cancer patients punctuate the otherwise lockstep assemblage of their cancer treatment decision-making with a persistent engagement in creative attempts to resist, thwart and otherwise manage the possibility of discrimination and likewise, the probability of institutional erasure in care settings. For many *Cancer’s Margins* interviewees, access to communicability of presence was thwarted by a widespread failure of cancer care environments (and providers) to engage in any way, whether in forms, educational pamphlets or provider-patient scripts, with LGBQ//T2 biographical narratives. Accordingly, a felt sense of unbelonging permeated contexts for cancer treatment, including access to culturally competent care and support groups. For folks who identify as transgender, service refusal and a lack of coordination of cancer care and gender affirming care were quotidian events.

The coordination and choreography of competing and oft-times incommensurable systems of knowledge -- including: biographical knowledge and biomedical knowledges as well as knowledge of gender affirming care and cancer care -- are left to the patient. Cancer care providers and organizations do not typically assist LGBQ//T2 patients to navigate health knowledge systems nor do they position those knowledge systems as pedagogically relevant to medical education. Given that current research on cancer health disparities within LGBQ//T2 populations (Cathcart-Rake 2018; Kamen et al. 2015; Polite et al. 2017) indicates greater cancer risks, lower screening rates and increased poor outcomes following treatment (Boehmer et al. 2013), it is essential that cancer surveillance systems collect data regarding sexual orientation and gender identity and that cancer care organizations customize the design of electronic health records and care protocols so as to meet the differentiated needs of sexual and/or gender minority cancer patients (see Beagan, Fredericks and Bryson 2015).

Population health models intended to signal under-investigated groups and that deploy signifying terms like “LGBT cancer” or “SGM cancer patients” address what are undoubtedly overlooked populations. However, these single variable models also tend to collapse gender and sexuality and to overlook intersectional complexities of cancer patients’ actual identities and histories. Our findings document how it is that both breast and gynecologic cancers and related treatments profoundly disrupt biographical knowledge concerning how LGBQ//T2 people narrate their altered bodies generally, and felt sense of gender and sexuality specifically. For a majority of our LGBQ interviewees, gender identity and expression were described in terms indicating an awareness of a felt sense of gender (e.g., butch, tomboy, femme) that is differentiated from biological sex (female). Gender identity and a sense of felt gender shape experiences of cancer treatment decision making, experiences of surgery and cancer induced changes during and after treatment. Our analysis of interviews with transgender folks illustrate that they encounter and navigate a system of breast and gynecological cancer care practises and knowledge utterly unprepared to include them. Trans cancer patients’ awareness of overlaps in cancer surgeries and gender affirming surgeries and of the lack of coordination of cancer and gender affirming care appears to be a problem of which care providers are woefully unaware.

Our findings bring critical attention to the demands that both cancer itself, and the systems of cancer health, place on LGBQ//T2 patients and the people in their social network. The design of *structurally* competent cancer health systems (and medical education) would entail attention to structural systems of knowledge and inequalities and permit clinicians to exceed the limitations of adaptations to differences misconstrued as individual cultural variations (Metzl and Hansen 2013). Communitarian, culturally-specific knowledge, rather than being marginalized as “anecdotal,” would instead be accorded the status of “experiential evidence” (Ziebland and Herxheimer 2008). Reductively gendered breast or gynecologic cancer knowledge regimes are designed in ways that, at best, discipline or undermine patient decision-making, and at worst, deny care when patients make decisions about their treatment based on understandings of self and embodiment that run counter to a normalized heterosexual cisgender femininity. The persistent lack of attention to LGBQ//T2 health in North American medical schools today (Obedin-Maliver et al. 2011) underscores the current relevance of longstanding claims of an urgent need for foundational evidence concerning sexual and gender minority cancer health. Our findings provide a partial answer to the growing need in Canadian and American public health generally, and cancer care specifically, for evidence that could inform the design of medically appropriate and culturally safe/r care. Cancer healthcare professionals, community-health organizations, and public health policy design would benefit, we hold, from a better understanding of how groups that are historically marginalized in health care settings and discourses negotiate an invariably complex choreography of health and care decision-making.

## Footnotes

1 Use of the LGBQ//T2 acronym does not imply a naive assumption about any kind of straightforward relationship between such arbitrary markers of identification and universal aspects of being, ontology, or essence. Rather, these deeply problematic, spatially and temporally situated and contested signifiers discursively mediate the visibility of groups and have both subjugatory and liberatory effects.

2 The bio info that accompanies each transcript quote includes a pseudonym and those markers that the speaker provided to signal a particular style of sexual and gender identification, age, race, type of cancer diagnosis, and location.
